# SAHA could inhibit TGF-β1/p38 pathway in MI-induced cardiac fibrosis through DUSP4 overexpression

**DOI:** 10.1007/s00380-021-01900-4

**Published:** 2021-07-08

**Authors:** Kaihao Wang, Ruijie Tang, Siyuan Wang, Wenyao Wang, Kuo Zhang, Jun Li, Ping Li, Yi-Da Tang

**Affiliations:** 1 Departments of Cardiology, State Key Laboratory of Cardiovascular Disease, National Center for Cardiovascular Diseases, Fuwai Hospital, Chinese Academy of Medical Sciences and Peking Union Medical College, Beijing, 100037 China; 2 Department of Cardiology and Institute of Vascular Medicine, Key Laboratory of Molecular Cardiovascular Science, Ministry of Education, Peking University Third Hospital, Beijing, 100191 China

**Keywords:** Cardiac fibrosis, Suberoylanilide hydroxamic acid (SAHA), TGF-β1, Dual-specificity phosphatase 4 (DUSP4)

## Abstract

Growing evidences have revealed that a histone deacetylase inhibitor (HDACi), suberoylanilide hydroxamic acid (SAHA) has anti-fibrotic effect in different diseases. In this study, we first evaluated whether SAHA could suppress cardiac fibrosis. Mice with MI-induced cardiac fibrosis were treated with SAHA by intraperitoneal injection and their cardiac function was improved after SAHA treatment. Results of western blotting and qRT-PCR in heart tissues suggested that TGFβ1/P38 pathway was activated in MI mice, and this effect was reversed by SAHA. Cell proliferation assay suggested that SAHA could suppress TGF-β1-induced cardiac fibroblasts proliferation. Furthermore, results of western blotting and qRT-PCR in cardiac fibroblasts depicted that SAHA may exert its anti-fibrotic effect through inhibiting TGF-β1-induced P38 phosphorylation by promoting DUSP4 expression. Our findings may substantiate SAHA as a promising treatment for MI-induced cardiac fibrosis.

## Introduction

Cardiac fibrosis is a worldwide problem which still lack efficacious treatment and is characterized by net accumulation of extracellular matrix (ECM, mainly containing Collagen 1 and Collagen 3) in myocardium [1]. Myofibroblasts are effector cells for cardiac fibrosis and characterized by appearance of α-smooth muscle actin (α-SMA) stress fibers [2, 3].

Evidence indicates that p38 mitogen-activated protein kinase (p38 MAPK) is a significant intracellular signaling pathway involved in cardiac remodeling and maladaptive processes post MI [4, 5]. Activation of p38 could cause proliferation, differentiation, secretion of collagen fibers in myocardial fibroblasts, ultimately leading to myocardial fibrosis [6].

Dual-specificity phosphatases (DUSPs) have recently drawn significant attention in cardiomyopathy, especially DUSP1 and DUSP4 [7]. Previous studies have proved DUSP4 gene deletion promoted p38 phosphorylation in heart tissues, while have no effect on JNK or ERK1/2 [7].

Histone deacetylases (HDACs), a class of epigenetic modification enzymes, could cause histones to wrap DNA more tightly and result in inhibition of gene transcription. Recent studies have shown that activation of HDACs was associated with pathologic cardiac remodeling and other cardiac abnormality [8]. Zolinza®, also known as Suberoylanilide hydroxamic acid (SAHA, a HDAC inhibitor), has been proved to have anti-fibrotic effect in both bleomycin-induced pulmonary fibrosis and chlorhexidine gluconate-induced peritoneal fibrosis in mice [9, 10]. However, the role SAHA and HDAC1 could play in cardiac anti-fibrotic effect has not been elucidated.

In this study, we firstly confirmed that SAHA significantly suppressed cardiac fibrosis and posed as a promising anti-fibrotic reagent for treating cardiac fibrosis. We explored whether the anti-fibrotic effect of SAHA is mediated by stimulating DUSP4 to inactivate p38 MAPK signaling pathway. And we employed a well-accepted myocardial infarction (MI) injury model and investigate on the role of HDAC1 in DUSP4/p38 pathway using HDAC1 siRNA in fibroblasts isolated from neonatal mice.

## Materials and methods

### Study animals and MI model constructing

Male C57BL5 mice were obtained from Charles River, China. Mice were anesthetized with isoflurane and placed on a heating pad (37 °C). The heart was exposed through a left thoracotomy. For sham group, mice underwent the same procedure without occlusion of the LAD. Mice were given SAHA solution at concentration of 15 mg/kg [9] or equal volume of DMSO by intraperitoneal injection once a day after MI surgery from day 2 to 28.

### Cardiac fibroblasts isolation, cell culture and treatment with TGF-β1

Primary cardiac fibroblasts were isolated from neonatal mouse hearts. Ventricles were isolated and washed in PBS medium and transferred to a solution of 0.8 mg/mL collagenase type II (Worthington Labs) with agitation for 40 min. The cells were collected after centrifugation and strained through a 70-μm filter, then plated on a 10-cm dish for 1 h.

Cardiac fibroblasts were cultured in Dulbecco's modified Eagle's medium (DMEM, Gibco, USA), supplemented with 100 U/ml penicillin, 100 mg/ml streptomycin, 2 mM L-glutamine, and 10% fetal calf serum, respectively. The cells were treated with DMSO only or 5 μM SAHA, with or without recombinant human TGF-β1 (5 ng/mL; Peprotech, USA, 100–21) for 48 h.

### Masson trichrome and Sirius red staining

On day 28 after MI surgery, mice were sacrificed and hearts were isolated. A transverse mid-section of left ventricle (LV) was fixed in 10% paraformaldehyde for subsequent sectioning. The slices then were stained with hematoxylin and eosin (H&E) or Masson or Sirius. Images were acquired using a microscope (Olympus). Infarct tissue area and fibrosis content were quantified using ImageJ software.

### Echocardiographic measurements

At the end of the 28 days treatment, echocardiography was performed using a GE Vivid 7 Dimension System (GE Vingmed Ultrasound, Horten, Norway) coupled with a M12 L linear (Matrix) array ultrasound transducer probe (5–13 MHz). The parameters were measured according to guidelines provided by manufacturer.

### Cell viability assay

4 × 103 mice cardiac fibroblasts were seeded per well in a 96-well plate. The cardiac fibroblasts viability was determined using Cell Counting Kit-8 (CCK8) (DOJINDO, Shanghai, China) according to the manufacturer’s instructions. CCK-8 reagent (10 µl) was added to each well and the plates were incubated at 37 ℃ for 3 h. Absorbance at 450 nm was determined with a microplate reader.

### HDAC1 activity assays

HDAC1 activity was measured using a HDAC1 colorimetric activity assay kit (GENMED SCIENTIFICS INC., USA, GMS50082.2.1 VA) following the manufacturer’s protocol. In brief, nuclear extracts of mouse cardiac fibroblasts were incubated with Color de Lys substrate at 37 ℃ for 60 min. Then Color de Lys developer was added to the samples and incubated at 37 ℃ for 15 min. Absorbance was measured in microplate reader measures at 405 nm. HDAC1 activity was calculated using the formula according to the manufacturer’s protocol.

### Determination of α-SMA-positive cells by flow Cytometry

Mouse cardiac fibroblasts were cultured in 6-well plates and treated with SAHA or TGF-β1. Then cells were fixed with 4% paraformaldehyde for 15 min and permeabilized with 0.5% Triton X-100 for 20 min. After being rinsed twice with PBS, cardiac fibroblasts were incubated with anti-α-SMA primary antibody (1:50, Abcam, ab124964) overnight at 4 °C. After being washed twice with PBS, cells were then incubated with Goat polyclonal Secondary Antibody to Rabbit IgG—H&L (Alexa Fluor® 594) (1:2000, Abcam, ab150080) for 2 h at 37 °C. After washing twice with PBS, cells were resuspended in 500 μl PBS and analyzed by flow cytometer (BD Biosciences, San Jose, USA). Cardiac fibroblasts stained with Alexa Fluor® 594 were α-SMA-positive cells.

### Cell transfection

Mouse cardiac fibroblasts were seeded and cultured in complete medium in 6-well plates. When cells reached 50–60% confluence, small interfering RNA (siRNA) oligonucleotides against DUSP4, HDAC1 (RIBOBIO, China) were transfected into mouse cardiac fibroblasts using Lipofectamine Max (Thermo Fisher Scientific, Inc.) in Opti-MEM^®^ Reduced Serum Medium (Thermo Fisher Scientifc, Inc.).

### Western Blotting

Proteins were extracted from cells or heart tissue samples. Protein concentrations were measured with the BCA assay (Beyotime, China). 25 μg lysate samples were separated on NuPage 4–12% Bis–Tris Gels (Novex, Life Technologies, CA, USA). The primary antibodies of TGF-beta 1 (1:1000; ab92486), HDAC1 (1:1000; ab109411), DUSP4 (1:1000, ab216576), P38 (1:1,000; ab170099), Phospho-P38 (1:1,000; ab195049) were purchased from Abcam. Histone H3 (1:1000, 9715S), Acetyl-Histone H3 (1:1000; 8173S), were purchased from Cell Signaling Technology. The secondary antibodies (1:5000) were purchased from Zhongshanjinqiao.

### Quantitative real-time PCR (qRT-PCR) for gene expression

Total RNA was extracted using TRIzol reagent followed by a RNA purification kit (Cat. 12183018A, ThermoFisher Scientific, Pittsburgh, PA) and DNase kit (Qiagen Inc., Valencia, CA). RNA (1 µg) was reverse transcribed to cDNA using RT2 First Strand Kit (Qiagen Inc., CA, USA) and then quantified by qRT-PCR (BIO-RAD, Cat. 1,725,124, USA). The primers are as following:

DUSP4, F 5’- CGTGCGCTGCAATACCATC—3’, R 5’- CTCATAGCCACCTTTAAGCAGG—3’;

Col1a1, F 5’-GCTCCTCTTAGGGGCCACT-3’, R 5’-CCACGTCTCACCATTGGGG- 3’;

Col3a1, F 5’-CTGTAACATGGAAACTGGGGAAA-3’, R 5’-CATAGCTGAACTGAAAACCACC-3’;

a-SMA,F 5’-GTCCCAGACATCAGGGAGTAA- 3’, R 5’-TCGGATACTTCAGCGTCAGGA- 3’;

GAPDH, F 5’-AGGTCGGTGTGAACGGATTTG-3’ R 5’-TGTAGACCATGTAGTTGAGGTCA-3’

### Statistical analysis

Continuous data are expressed as mean ± SD. To test if it is statistically significant different between two groups, we used the unpaired two tailed Student’s t test. To test if it is statistically significant different between multiple comparisons we used one-way ANOVA with Bonferroni correction. GraphPad Prism 7.0 statistical software was used to analyze the data. Significance was accepted at *P* < 0.05.

## Results

### SAHA improved MI-induced cardiac fibrosis in mice

**Fig. 1:**
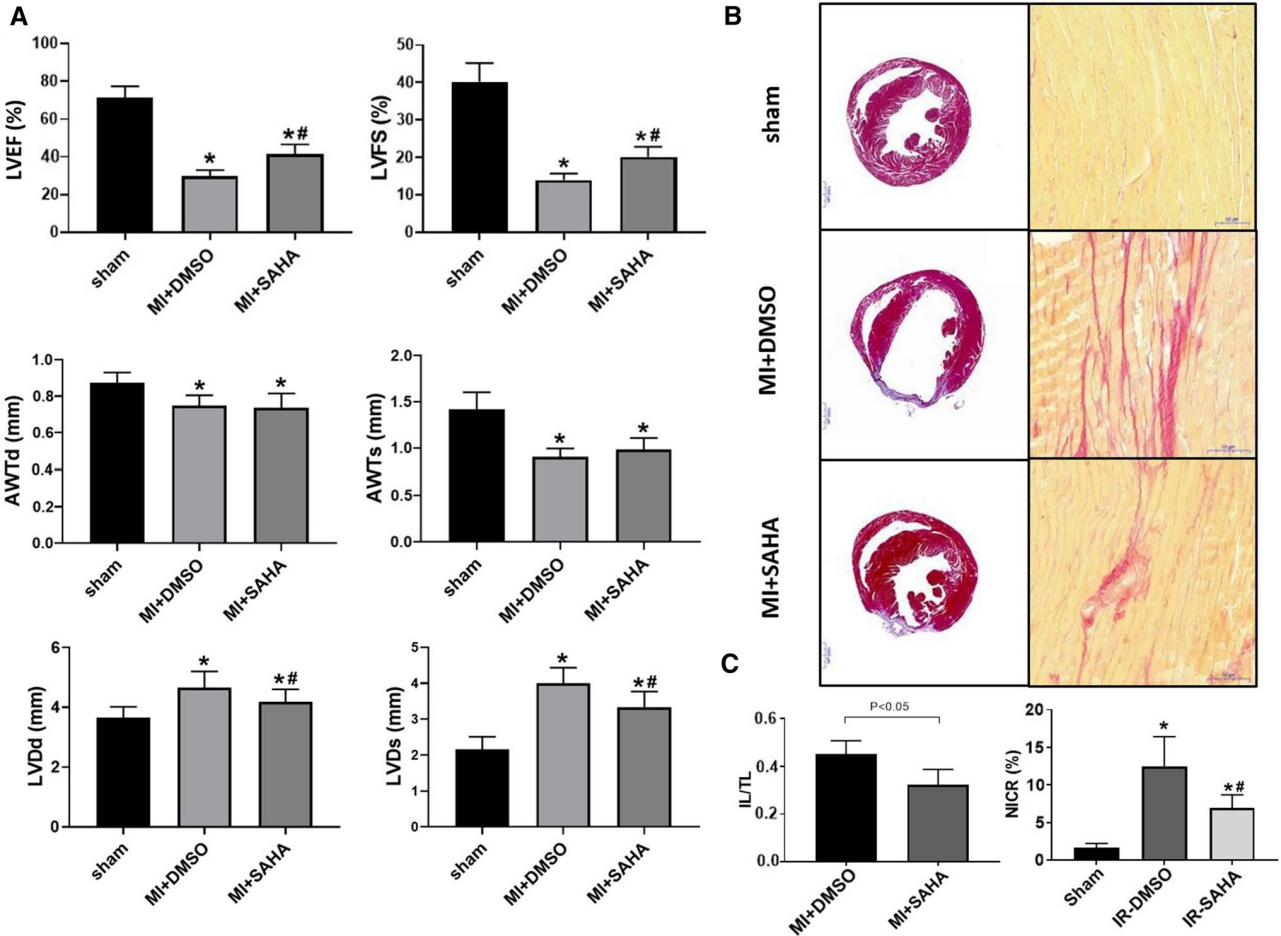
Treatment of SAHA improved cardiac function in mice after MI **a** Echocardiographic analysis of sham mice, MI + DMSO mice and MI + SAHA mice. LVEF (left ventricular eject fraction), LVFS(left ventricular fractional shortening), AWTd (left ventricular anterior wall thickness in diastole), AWTs(left ventricular anterior wall thickness in systole), LVDd(left ventricular diameter in diastole) and LVDs(left ventricular diameter in systole) were calculated according to the guidelines. N = 12 for all groups. One-way ANOVA with Tukey’s post-hoc multiple-group comparisons was used in statistical analysis *p < 0.05 vs. Sham, #p < 0.05 vs. MI + DMSO. **b** Myocardial infarction size assessed by Masson staining and Sirius Red. **c** IL(infarction length)/TL(total cross-sectional left heart length) and NICR(collage ratio in non-infarct area) were analyzed. Unpaired two tailed student’s t test comparison was used for statistical analysis. Bar graphs show group means ± SD. *N* = 6 for each group. One-way ANOVA with Tukey’s post-hoc multiple-group comparisons was used in statistical analysis *p < 0.05 vs. Sham, #*p* < 0.05 vs. MI + DMSO.

To investigate into the role of SAHA in MI, myocardial MI was performed on C57BL6 mice followed by SAHA treatment. Cardiac function deteriorated after MI surgery, as LVEF (71.4 ± 5.9% vs. 29.9 ± 3.2%, p < 0.05) and LVFS (40.2 ± 5.0% vs. 14.0 ± 1.7%, *p* < 0.05) decreased compared to sham group. However, both LVEF and LVFS were significantly improved after treatment of SAHA. LV internal diameter in systole (LVDs) was increased in MI + DMSO group compared with sham (4.00 ± 0.44 mm vs. 2.16 ± 0.34 mm, *p* < 0.05) and decreased significantly to 3.34 ± 0.43 mm in MI + SAHA group (Fig. 1a).

To further test the effect of SAHA on cardiac fibrosis, we used Masson Trichrome and Sirius Red staining to test fibrosis area on Sham, MI + DMSO and MI + SAHA mice groups. Masson Trichrome staining showed MI surgery caused obvious cardiac remodeling while SAHA treatment reduced myocardial infarction area compared with MI + DMSO group (Fig. 1b). Sirius Red staining further revealed that the collagen deposition increased in MI + DMSO group in non-infarcted zone (12.5 ± 4.0% in MI + DMSO group vs. 1.6 ± 0.6% in sham group, p < 0.05) compared to sham group. And the collagen deposition obviously decreased in MI + SAHA group compared with MI + DMSO group (Fig. 1b). Besides, we also found that infarct length/ total cross-sectional left heart length (IL/TL) and NICR(collage ratio in non-infarct area) decreased after SAHA treatment compared with MI + DMSO group (Fig. 1c).Taken together, these data indicated that SAHA could attenuate MI-induced cardiac dysfunction and cardiac fibrosis.

### SAHA prevented MI-induced P38 phosphorylation and ECM generation

**Fig.2:**
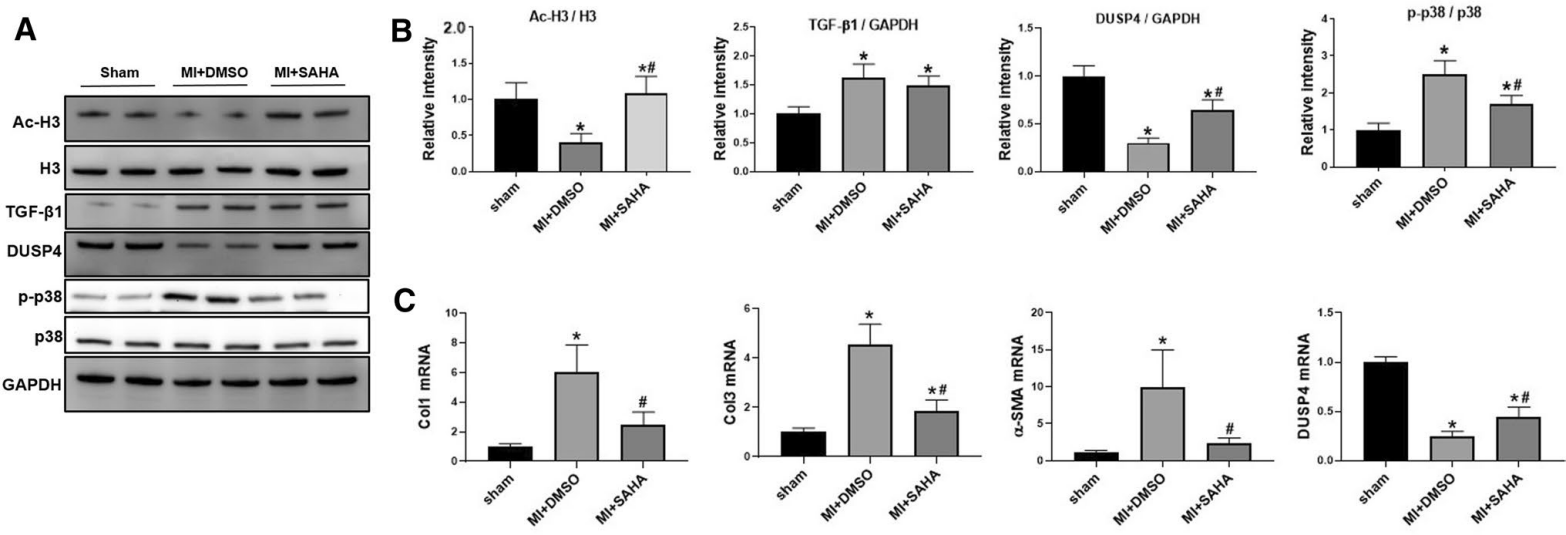
Treatment of SAHA reversed effect of P38 activation and ECM generation induced by MI in mice **a** Protein level of Ac-H3, DUSP4, TGF-β1 and p-p38 analyzed by Western blotting in each group of heart tissue. **b**The intensity of the blots for DUSP4, Ac-H3/H3, TGF-β1 and p-p38/p38, standardized by GAPDH. **c** Expression of Col1a, Col3a, α-SMA and DUSP4mRNA in heart tissue analyzed by qRT-PCR. *N* = 6 for each group. One-way ANOVA with Tukey’s post-hoc multiple-group comparisons was used for statistical analysis. *p < 0.05 vs. Sham, #*p* < 0.05 vs. MI + DMSO. Bar graphs show group mean ± SD.

To further investigate on the mechanism of cardiac function improvement by SAHA, we used western blotting to detect protein expression of Acetyl-Histone H3 (Ac-H3), DUSP4, TGF-β1 and Phospho-P38 (p-p38) in mice heart tissues. The protein level of Histone H3 (H3) had no obvious changes between groups. However, Ac-H3 decreased in mice post MI compared with sham group (0.41 ± 0.12 vs. 1.00 ± 0.23, p < 0.05) while SAHA treatment significantly increased Ac-H3 expression in MI mice. Besides, the protein level of TGF-β1 was significantly increased after MI and there was no obvious difference between MI + DMSO and MI + SAHA group. DUSP4 expression level decreased in MI mice and SAHA treatment significantly increased its expression compared to MI + DMSO group, (0.30 ± 0.05 in MI + DMSO group vs. 0.64 ± 0.10 in MI + SAHA group, *p* < 0.05). Besides, the p38 phosphorylation level was significantly increased in MI + DMSO group compared with Sham group, while SAHA significantly reduced the level of p-p38 (2.50 ± 0.38 in MI + DMSO group vs. 1.69 ± 0.24 in MI + SAHA group, *p* < 0.05) (Fig. 2a , b).

Furthermore, we detected mRNA expression level in heart tissues to investigate on downstream genes of TGF-β1/p38 pathway, including Collagen 1 (Col1), Collagen 3(Col3) and α-SMA. qRT-PCR results showed MI exposure greatly increased the expression of Col1a, Col3a and α-SMA mRNA while SAHA treatment significantly reduced the expression level of Col1, Col3 and α-SMA mRNA in mice post MI (Fig. 2c). These data demonstrated that SAHA could attenuate fibroblasts genesis and ECM deposition induced by MI. Besides, DUSP4 mRNA level obviously decreased after MI surgery (0.25 ± 0.05 in MI + DMSO group vs. 1.00 ± 0.05 in sham group, *p* < 0.05) and increased after SAHA treatment in MI mice (0.46 ± 0.03 in MI + SAHA group vs. 0.25 ± 0.05 in MI + DMSO group) (Fig. 2c). This gave us a clue that increased DUSP4 expression may contribute to reduced phosphorylation of p38 in MI mice with SAHA treatment.

### SAHA could inhibit TGF-β1/P38 pathway in cardiac fibroblasts

**Fig.3:**
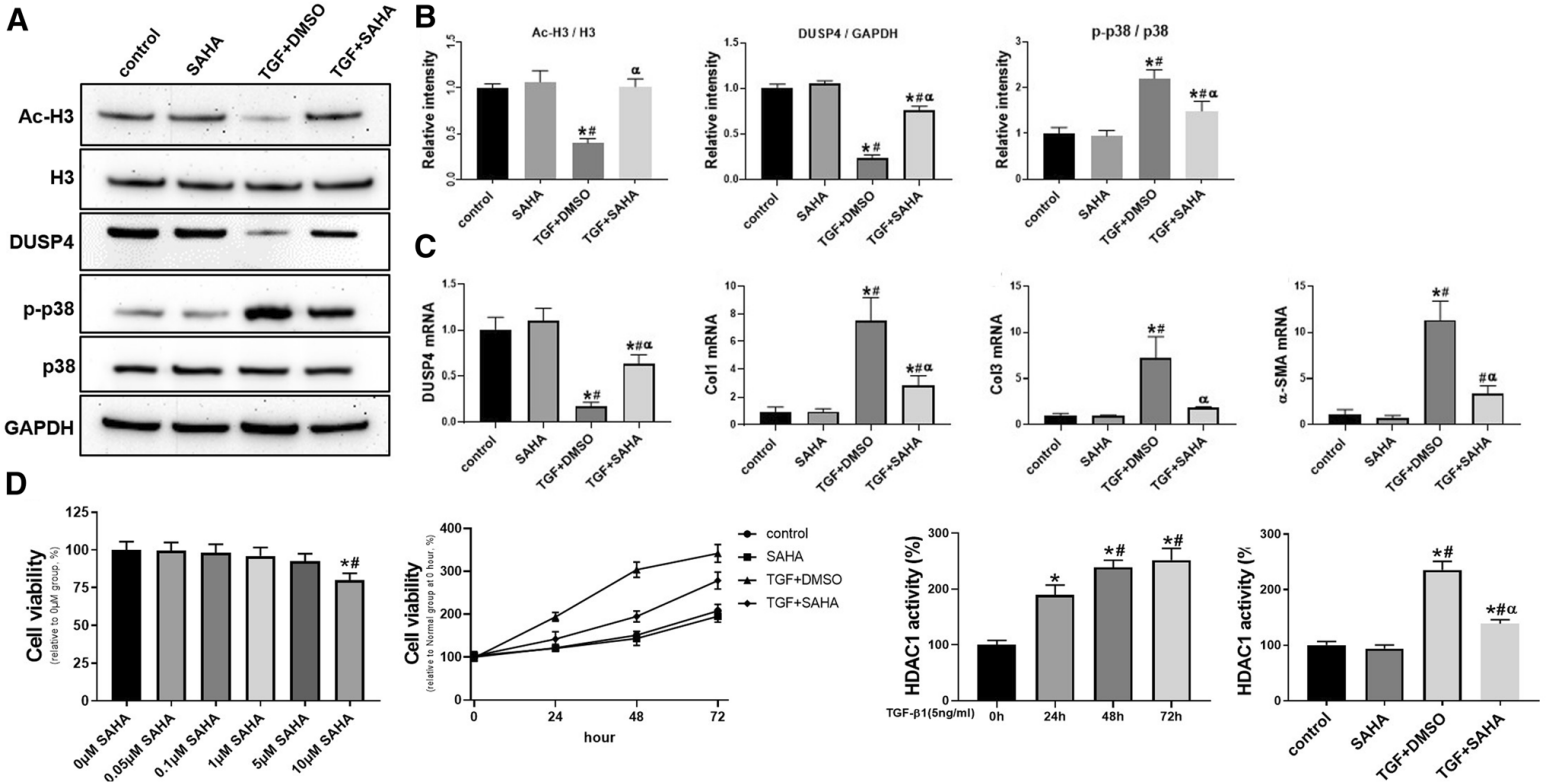
SAHA treatment could inhibit TGF-β1-induced cell proliferation expression, P38 phosphorylation and ECM generation in cardiac fibroblasts **a** Protein level of Acetyl-Histone H3, DUSP4, TGF-β1 and p-p38 analyzed by Western blotting in fibroblasts. **b** Intensity of the blots for Ac-H3/H3, DUSP4, and p-p38/p38standardized by GAPDH; **c** Expression of Col1a, Col3a, α-SMA and DUSP4 mRNA in fibroblasts analyzed by qRT-PCR; N = 4 for each group. One-way ANOVA with Tukey’s post-hoc multiple-group comparisons was used for statistical analysis. *p < 0.05 vs. control, #*p* < 0.05 vs. only SAHA, αp < 0.05 vs. TGF + DMSO group. **d** Cell viability changes induced by SAHA for cardiac fibroblasts analyzed by CCK-8 assay. **e** HDAC1 activity changes induced by SAHA for cardiac fibroblasts analyzed by Results was expressed as percentage of activity compared to control group (no TGF- β1 and no SAHA). *N* = 4 for each group. One-way ANOVA with Tukey’s post-hoc multiple-group comparisons was used for statistical analysis. *p < 0.05 vs. Sham, #*p* < 0.05 vs. only SAHA group.

To further test the impact of SAHA treatment on TGF-β1/P38 pathway, cardiac fibroblasts isolated from neonatal mice were pretreated with TGF-β1 (5 ng/mL) and then rescued with SAHA (5 μM). Western blotting results showed that TGF-β1 can significantly reduce Ac-H3 and DUSP4 protein levels compared with control group. Meanwhile, the phosphorylation of p38 in cardiac fibroblasts was significantly increased with TGF-β1 treatment compared with control group (2.20 ± 0.19 in TGF + DMAO group vs. 1.00 ± 0.13 in control group, *p* < 0.05). Compared with TGF + DMSO group, TGF + SAHA treatment significantly increased the level of Ac-H3 and DUSP4, while significantly decreased ratio of p-p38/p38 (1.49 ± 0.21 in TGF + SAHA group vs. 2.20 ± 0.19 in TGF + DMSO group, *p* < 0.05) (Fig. 3a, b). There was no significant difference in DUSP4 and p-p38 between control and SAHA group.

Next, we detected Col1, Col3 and α-SMA mRNA levels in fibroblasts. qRT-PCR results showed that SAHA blocked TGF-β1-induced overexpression of Col1 and Col3 mRNA expression. Besides, TGF-β1 promoted α-SMA mRNA expression while SAHA significantly reduced α-SMA mRNA. And the reduction of DUSP4 mRNA induced by TGF-β1 was reversed by SAHA treatment (0.75 ± 0.05 in TGF + SAHA group vs. 0.24 ± 0.03 in TGF + DMSO group, *p* < 0.05) (Fig. 3c).

CCK-8 assay was used to detect the effect of SAHA on fibroblasts. Results showed that 10 µM SAHA decreased cell viability to 80.00 ± 4.55% (P < 0.05). However, 0.05, 0.1, 0.5,1 and 5 µM showed no detectable toxicity. Thus, we used 5 µM SAHA for the following detection. Cardiac fibroblasts were divided into four groups: control, SAHA group, TGF + DMSO group and TGF + SAHA group. Results showed that the cell density increased much slower in TGF + SAHA group than in TGF + DMSO group (Fig. 3d). There is no obvious difference between control and SAHA group. These data demonstrated TGF-β1-induced proliferative process of cardiac fibroblasts was inhibited by SAHA.

Then we measured HDAC1 activity in TGF-β1-treated (5 ng/ml, 72 h) myocardial fibroblasts. TGF-β1 significantly increased HDAC1 activity (Fig. 3e) in a time-dependent manner, as HDAC1 activity was significantly higher at 48 h than 24 h. Then cardiac fibroblasts were divided into four groups: control, SAHA group, TGF + DMSO group and TGF + SAHA group. HDAC1 activity in TGF + DMSO group is much higher than in control group and SAHA treatment significantly reduced HDAC1 activity (235.5 ± 15.3% in TGF + DMSO group vs. 138.6 ± 7.3% in TGF + SAHA group). These data demonstrated that SAHA treatment could inhibit TGF-β1-induced HDAC1 activity.

### SAHA inhibited TGF-β1/P38 pathway in cardiac fibroblasts by upregulating DUSP4

**Fig. 4:**
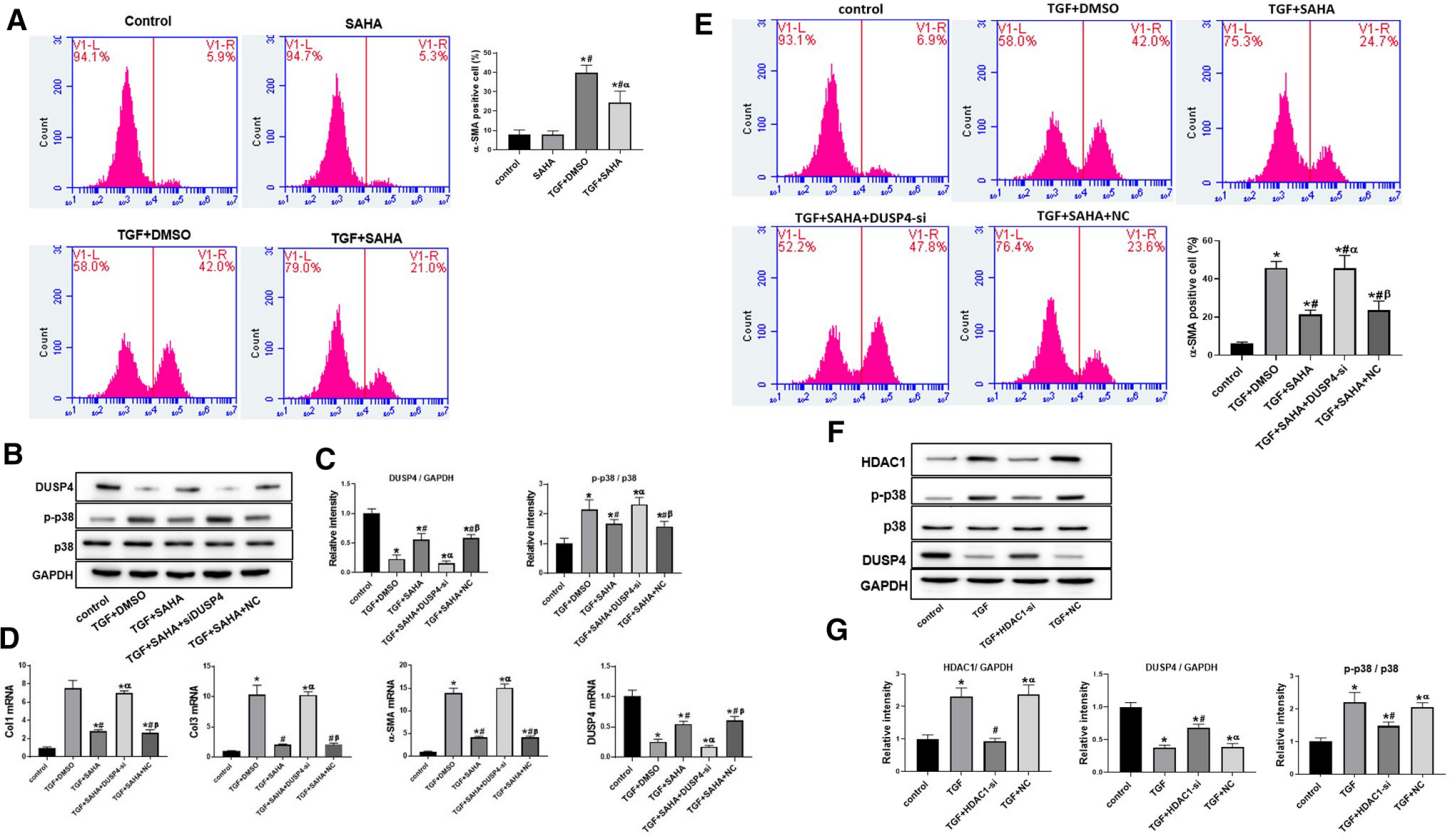
SAHA inhibited TGF-β1/p38 pathway and α-SMA expression by increasing DUSP4 expression in fibroblasts **a** α-SMA expression and calculated α-SMA-positive rate of myocardial fibroblasts analyzed by flow cytometry in each group. Bar graphs show group means ± SD. *N* = 4 for each group. **b** Protein level of DUSP4 and p-p38 analyzed by Western blotting in each group in fibroblasts. **c** Intensity of the blots for DUSP4 and p-p38/p38standardized by GAPDH; **d** Expression of Col1a, Col3a, α-SMA and DUSP4 mRNA in fibroblasts analyzed by qRT-PCR. One-way ANOVA with Tukey’s post-hoc multiple-group comparisons was used for statistical analysis. *p < 0.05 vs. control, #*p* < 0.05 vs. only SAHA, α*p* < 0.05 vs. TGF + DMSO group. β*p* < 0.05 vs. TGF + SAHA + DUSP4-si group; NC: siRNA negative control. **e** α-SMA expression and α-SMA-positive rate in fibroblasts analyzed by flow cytometry in each group. Bar graphs show group means ± SD. **f **Protein level of HDAC1, DUSP4 and p-p38 analyzed by Western blotting in each group in fibroblasts. **g** Intensity of the blots for HDAC1, DUSP4 and p-p38/p38standardized by GAPDH. *N* = 4 for each group. One-way ANOVA with Tukey’s post-hoc multiple-group comparisons was used for statistical analysis. *p < 0.05 vs. control, #*p* < 0.05 vs. TGF, α*p* < 0.05 vs. TGF + HDAC1-si; NC: siRNA negative control.

The conversion of myocardial fibroblasts to myofibroblasts is essential in the process of myocardial fibrosis [11]. a-SMA is an important marker of this differentiation process [12]. Flow cytometry was used to detect differentiation of cardiac fibroblasts after SAHA treatment. Results showed that the proportions of a-SMA-positive cells were much higher in TGF + DMSO group compared with control group. SAHA treatment significantly reduced the proportion of myofibroblasts induced by TGF-β1 (39.9 ± 3.7% in TGF + DMSO group vs. 24.4 ± 5.9% in TGF + SAHA group, *p* < 0.05) (Fig. 4a). No difference was observed in proportion of myofibroblasts between control and SAHA group.

Then we detected the protein and mRNA level in SAHA treated fibroblasts. As shown in Fig. 4b, SAHA can upregulate DUSP4 and down-regulate p-p38 expression. Small interfering RNA (siRNA) oligonucleotides against DUSP4 was used to knockdown DUSP4 expression. We found that DUSP-si reversed the reduction of p38 phosphorylation by SAHA (p-p38, 2.32 ± 0.24 in TGF + SAHA + DUSP4-si group vs. 1.68 ± 0.14 in TGF + SAHA group, P < 0.05) (Fig. 4b, c). qRT-PCR results showed that SAHA blocked TGF-β1-induced overexpression in col1a, col3a, α-SMA. And these mRNA levels were significantly reversed by siRNA against DUSP4 (Fig. 4d).

Furthermore, results of flow cytometry depicted that SAHA significantly suppressed the proportion of α-SMA-positive cells in TGF + SAHA, compared with the TGF + DMSO group. And this effect was significantly abrogated by DUSP-si (21.35 ± 2.34% in TGF + SAHA group vs. 45.63 ± 6.73% in the TGF + SAHA + DUSP4-si group, *P* < 0.05; Fig. 4e).

To investigate into whether the increased expression of DUSP4 is related to HDAC1, we used HDAC1-si to knock down HDAC1 mRNA in cardiac fibroblasts, then DUSP4, p-p38 protein levels were analyzed in TGF-β1 treated cells. Compared with TGF + NC group, DUSP4 protein expression was significantly increased in TGF + HDAC1-si group (0.68 ± 0.05 in TGF + HDAC1-si group vs. 0.38 ± 0.06 in TGF + NC group, *P* < 0.05). Besides, increased p38 phosphorylation level induced by TGF-β1 was abolished by HDAC-si (2.05 ± 0.14 in TGF + NC group vs. 1.47 ± 0.12 in TGF + HDAC1-si group, *P* < 0.05) (Fig. 4f, g).

## Discussion

Excessive fibrous tissue proliferation after injury can lead to myocardial fibrosis, resulting in impaired heart function and heart failure [13, 14]. Effective treatment to inhibit myocardial fibrosis after myocardial infarction are of significant importance.

Previous studies have shown HDAC inhibition had anti-fibrotic effect in various diseases, including renal fibrosis [15], pulmonary Fibrosis [8, 16] and liver fibrosis [17]. However, whether SAHA, as a broad-spectrum histone deacetylase inhibitor, can reduce myocardial fibrosis has not been well understood. In this study, we firstly focused on the effect and mechanism of SAHA on MI-induced cardiac fibrosis. Then we proved that SAHA could reduce infarction area in MI model mice.

SAHA, as a HDAC inhibitor, could increase the accumulation of hyperacetylated histones H3, directly influencing chromatin structure and, thereby, the relationship of the nucleosome and the gene promoter elements [18]. Histone acetylation reduces the binding between histones and DNA, leading to a more open structure which is more accessible to the transcriptional machinery. In our study, TGF-β1 reduced Ac-H3 and DUSP4 protein levels while SAHA treatment could significantly reverse the level of Ac-H3 and DUSP4, indicating that SAHA could reverse TGF-β induced low acetylation level.

Various evidence demonstrated the important role of p38 MAPK played in cardiac fibrosis [6]. The p38 pathway is important for upregulating the expression of specific proteins in cardiac fibroblasts, including matrix metalloproteinases (MMPs), α-smooth muscle action (α-SMA) [19–21]. Thus inhibiting p38 pathway may therefore be a potential therapy to ameliorate MI-induced myocardial remodeling [22]. Our study proved that the p-p38/p38 ratio increased in mice hearts post MI, while SAHA significantly suppressed the process. Besides, SAHA could inhibit the increase of p38/p38 levels after treating with TGF-β1. The expression of DUSP4 was significantly up-regulated after SAHA treatment in mice myocardial tissues post MI. Multiple studies have shown that activating DUSPs can inhibit cardiac remodeling and cardiac fibrosis [23–25]. DUSP4 degradation promoted p38 activation, leading to renal fibrosis [26] and endometrial fibrosis [27]. Our studies revealed that anti-fibrotic effects of SAHA on cardiac fibroblasts were largely abolished by transfection with siRNA against DUSP4, indicating that DUSP4 could mediate anti-fibrotic process of SAHA on mice cardiac fibroblasts.

Taken together, this study may be the first report of positive regulation of DUSP4 by SAHA in cardiac fibrosis. By promoting the expression of DUSP4, SAHA could block TGF-β1/p38 signaling, thus inhibiting myofibroblast formation and cardiac fibrosis, suggesting that SAHA could be a promising anti-fibrotic reagent. However, more detailed mechanisms on how HDAC1 mediates DUSP4 expression remains to be explored in the future.
